# Effect of Levan Supplement in Orange Juice on Weight, Gastrointestinal Symptoms and Metabolic Profile of Healthy Subjects: Results of an 8-Week Clinical Trial

**DOI:** 10.3390/nu4070638

**Published:** 2012-07-04

**Authors:** Eva Niv, Yami Shapira, Ira Akiva, Evgenia Rokhkind, Etty Naor, Mira Arbiv, Nachum Vaisman

**Affiliations:** The Unit of Clinical Nutrition, Tel-Aviv Sourasky Medical Center, Tel-Aviv, 76589, Israel; Email: yamis@tasmc.health.gov.il (Y.S.); iraa@tasmc.health.gov.il (I.A.); evgeniar@tasmc.health.gov.il (E.R.); ettyn@tasmc.health.gov.il (E.N.); Miraa@tasmc.health.gov.il (M.A.); nachumv@tasmc.health.gov.il (N.V.)

**Keywords:** dietary fibers, levan, fructans, orange juice

## Abstract

Levan is a commonly used dietary fiber of the fructans group. Its impact on health remains undetermined. This double blind controlled study aimed to investigate the effect of 8 weeks’ daily consumption of 500 mL of natural orange juice enriched with 11.25 g of levan compared to the same amount of natural orange juice without levan on weight, gastrointestinal symptoms and metabolic profiles of 48 healthy volunteers. The statistical analyses compared between- and within-group findings at baseline, 4 weeks and study closure. The compared parameters were: weight, blood pressure, blood laboratory tests, daily number of defecations, scores of stool consistency, abdominal pain, bloating, gas, dyspepsia, vomiting and heartburn. Despite a higher fiber level recorded in the study group, there was no significant difference in the effect of the two kinds of juices on the studied parameters. Both juices decreased systolic and diastolic pressures, increased sodium level (within normal range), stool number, and bloating scores, and decreased gas scores. In conclusion*,* levan itself had no effect on weight, gastrointestinal symptoms or metabolic profile of healthy volunteers. Its possible effect on obese, hypertensive or hyperlipidemic patients should be investigated in further studies.

## 1. Introduction

Levan belongs to a group of fructans, which are biopolymers of fructose and composed of fructofuranosyl residues joined by β(2,6) and β(1,6) linkages. There are two main natural fructans, inulin and levan, which are distinguished by the type of linkage. Levan can be found in plants and in the bio-products of microorganisms. The length of levan varies from ten to thousands of fructose residues [1]. One advantage of fructose polymers is their ability to be fermented by gut microflora which, in turn, improves the intestinal flora [2] and increases mineral absorption [3,4]. Prebiotic fibers, such as inulin, pass through the small intestine without being digested and are fermented in the large intestine. Dietary inulin and other fructo-oligisaccharides have been shown to markedly increase bifidobacterium and lactobacillus in healthy humans and animals [5]. The prebiotic nature of levan is less understood. Several groups have studied the prebiotic effects of levan in a rat model and showed that total fecal weight, short-chain fatty acid production and the total number of microorganisms and acid-producing bacteria increased significantly with its use [6]. The hypocholesterolemic effect of levan was also studied in a rat model and was found to have a positive effect on weight and plasma lipids [7]. Finally, levan was shown to have an immunostimulatory effect, as demonstrated by the induction of IL-12 production and suppression of T-helper type 2 response and IgE production [8]. The effect of Levan on humans was largely not investigated in controlled studies till now. The only controlled study regarding the metabolic effect of Levan was conducted by Kang *et al.* in 2003 on 29 Korean women [9]. This study demonstrated that an addition of 6 g of Levan was effective in controlling weight, body fat, cholesterol profile and triglyceride levels. 

Levan is naturally present in various food products and regularly consumed by humans. Its content in food can be increased by applying fermentation techniques. “Natto” is a traditional Japanese food made of fermented soybeans, which contains high concentrations of levan that are produced during the fermentation process. Its concentration can also be increased by the enzyme, levansucrase. In juices, levansucrase can hydrolyze naturally occurring sucrose into glucose and fructose, and polymerize the obtained fructose into levan. By means of these processes, the amount of sucrose in a fruit juice can be reduced and the amount of levan (a dietary fiber) can be increased. These changes are very important for the dietary industry, especially in an era of functional food. 

Natural orange juice has a very high percent of sucrose as a natural component. The aim of this study was to compare the effect of 8 weeks of administration of natural orange juice fermented with levansucrase (reduced sugar percentage and increased fiber content) with that of regular natural orange juice on the weight, gastrointestinal symptoms and metabolic profile of healthy volunteers. 

## 2. Materials and Methods

This was a double blind parallel controlled study of healthy volunteers. It was conducted in the Unit of Clinical Nutrition of Tel-Aviv Sourasky Medical Center, Israel (Registered under ClinicalTrials.gov Identifier no. NCT00123456). The study design was approved by the local Helsinki Committee and all participants gave written informed consent. The subjects were recruited by newspaper advertisements and received financial compensation. They all underwent an initial screening evaluation, which included: medical history, medical examination, weight and blood pressure measurements, a 3-day food diary in order to establish their dietary habits, baseline stool and gastrointestinal questionnaires, blood tests for glucose, HbA1C, urea and creatinine, uric acid, GOT, GPT, GGT, alkaline phosphatase, total cholesterol, LDL and HDL cholesterol, triglycerides, total bilirubin, iron, transferrin, ferritin, sodium, potassium, chloride, calcium, magnesium, inorganic phosphate, zinc ,albumin, TSH and complete blood count. 

The inclusion criteria were: healthy volunteers aged 18–60 years, written informed consent, ability to drink orange juice and regular eating patterns. The exclusion criteria were: chronic disease (diabetes, cancer, chronic obstructive pulmonary disease, metabolic syndrome, overweight, kidney failure, heart disease and osteoporosis), antibiotic treatment during the past 4 weeks, taking regular probiotic or prebiotic supplementation (unless a 2-week pre-study washout is carried out), intestinal disorders (inflammatory bowel disease, irritable bowel disease, celiac, colon cancer), intestinal surgery, use of medications for lowering cholesterol and/or sugar control, pregnancy, abnormal baseline blood test results (liver and/or kidney function, total cholesterol >250 mg/dL and fasting glucose >120 mg/dL).

The consecutive enrollees were divided into two groups by double blind randomization (1:1.5). Those in the control group were assigned to drink 500 cc of regular fresh pasteurized orange juice daily for 2 months, while those in the study group drank the same amount of orange juice that was reduced in sugar and contained 11.25 g of levan (Table 1). Specifically, the latter juice contained 5 g/L of sucrose compared with 44 g/L in regular juice, and a higher glucose and sodium content (39 *versus* 21 g/L and 53 *versus* 9 mg/mL, respectively) produced during the fermentation. Both juices were produced by Gan Shmuel Factory, Foods, Ltd., Israel. Both juices came in the same packings, same bottles (but labeled as A or B), have the same texture and a similar taste. All participants were asked to adhere to their regular diet. Because of levan consumption, the study subjects consumed 11.25 g of added fibers to their previous diet. 

**Table 1:** Comparison between regular juice and levan-enriched juice.

	Juice with levan	Regular juice
Brix by refractometer	11.3	11.3
Acidity as anhydrous citric acid (g/100 mL)	0.72	0.75
Brix/acid ratio	16.5	16
pH	3.65	3.87
Vitamin C (mg/mL)	37	40
Potassium (mg/L)	1700	1850
Sodium (mg/mL)	53	9
Formol number	19.7	23.5
Glucose (g/L)	39	21
Fructose (g/L)	22	23
Sucrose (g/L)	5	44
% of sugars in fruit juice drink	6.3	8.4
Calculated levan content (g/L)	22.5	0
Other fibers (g/L)	3	3

All participants were asked to fill out a daily stool and gastrointestinal status questionnaire throughout the study period. The following items were included: compliance to juice consumption, number of defecations, and stool consistency and color. They were requested to score the intensity of abdominal pain, bloating, gas, dyspepsia, and report the presence of vomiting or heartburn. They were also asked to attend the Unit of Clinical Nutrition every two weeks for weight and blood pressure measurements, medical check-ups and interviews about compliance, general feeling and adverse events. Blood was drawn in the middle and the end of the study period (week 4 and week 8), and the above-cited tests were run. A telephonic interview was conducted weekly. At study closure, the subjects filled in a satisfaction questionnaire regarding the juice they had drunk as well as a 3-day food diary to ensure that there had been no changes in their regular diets. 

In addition to the study and control groups, five volunteers were asked to drink standard and levan-supplemented juice with an interval of several days in between juices in order to determine the glycemic index of each juice. They drank the specified juice after an overnight fast of 12 h, and blood was drawn for insulin and glucose at intervals of 0, 30, 60, 90 and 120 min after juice consumption. The areas under the glucose and the insulin curves were calculated and compared. Each subject was his/her own control. 

Selected parameters were analyzed statistically and compared between the study and control groups and within each group at the beginning, middle and end of the 8-week study period. The parameters were: body mass index (BMI), systolic and diastolic blood pressure, blood laboratory tests (chemistry, complete blood count, liver enzymes, lipid profile, HBA1C, iron parameters, TSH), as well as daily number of defecations, scores of stool consistency, and the presence and severity of any existing abdominal pain, bloating, gas, dyspepsia, vomiting and heartburn. The data of the study group were analyzed by one-way analysis of variance with repeated measures (points in time) using the mixed model. Pair-wise comparisons between time points were performed using Hochberg’s correction whenever the effect of time was found significant. When the interaction between study group and time was significant, it was sliced to test for simple effects for each group and time point. Values of *p* < 0.05 were considered statistically significant. In order to compare these parameters between the study and control groups and to examine changes over time, the data were first averaged over each week and then grouped into three time intervals, *i.e.*, week one (baseline), weeks 3–6, and weeks 7–8. Comparisons between areas under the glucose and insulin curves for evaluation of the glycemic index were performed using the paired two-tailed *t*-test. Each participant was used as his/her own control. All statistical analyses were performed using SAS for windows 9.2.

## 3. Results

Twenty-four of the 74 volunteers were not enrolled at the time of recruitment: 6 were not interested after hearing the details of the study, 15 had abnormal blood tests, one had a chronic disease, one does not like drink juice and one did not have regular eating habits. Another two subjects were dropped shortly after study entry due to non-compliance, leaving a total of 48 subjects. They were randomly divided into two groups: 19 drank 500 cc of regular fresh pasteurized orange juice every day for 8 weeks (controls) and 29 drank sugar-reduced orange juice that contained 11.25 g of levan. The demographics of the study population are presented in Table 2. The self-reported baseline fiber consumption in both groups was similar: 11.2 g in the study group *versus* 13 g in the control group (*p* = 0.135). On baseline the participants did not drink fruit juice on a daily basis but occasionally only. Three-day food diary at the closure of the study confirmed that the participants did not perform changes in the regular diet (as they were asked not to do). 

**Table 2:** Demographics of the study population.

	Regular juice group	Juice with levan group	*p* value
Number of subjects	19	29	
Gender M/F	8/11	15/14	
Average age (years)	35.36 ± 7.8	37.62 ± 9.7	NS
Body mass index	25.09 ± 2.3	24.53 ± 2.5	NS
Fibers (g/day)	13 ± 1.4	11.2 ± 2.1	NS

The results of the mixed model for relative changes in the gastrointestinal status during the 8-week study period are presented in Table 3. Drinking orange juice caused a significant increase in the number of stools per week in both groups (*p* = 0.02) on expense of an increase in semi-solid and fluid stools (*p* = 0.023 and 0.01, respectively). There was no difference in the number of stools per week between the two groups (*p* = 0.79). In addition, consumption of orange juice increased the number of complaints of abdominal bloating and decreased the gas scores (*p* = 0.05 and 0.01, respectively) in both control and study groups (*p* = 0.39 and 0.93, respectively). There were no between- or within-group differences over time with regard to other gastrointestinal symptoms (abdominal pain, vomiting, heartburn and epigastric pain). 

The drinking of regular or levan-enriched sugar-reduced juice over 8 weeks produced minimal if any effects on metabolic profiles and blood tests (Table 4). BMI, glucose, HbA1C, cholesterol (both LDL and HDL), and triglyceride levels underwent no between- or within-group changes. Both systolic and diastolic pressures decreased significantly after the regimen of orange juice drinking was initiated (*p* = 0.04 and 0.03, respectively), but with no differences between the two groups (*p* = 0.27 and 0.67, respectively). Both the baseline and the endpoint blood pressure were normal in both groups. The sodium level increased significantly during the study in both groups (*p* = 0.009), but with no difference between the groups (*p* = 0.38), and still remaining within the normal range. Statistical analysis showed transient changes in creatinine and bilirubin levels (within normal limits), but both levels returned at week 8 to the baseline levels. Calcium levels decreased significantly at week 4 (within normal limits) compared to baseline in both groups, and returned to original levels at week 8 (*p* = 0.0005) for both groups (*p* = 0.91). The rest of the blood tests were not influenced by the consumption of either type of orange juice, nor were there any adverse effects. 

The glycemic index of both types of juices was examined in five volunteers (Table 5). The area under the insulin curve tended to be lower in the levan-enriched group compared to the regular juice (*p* = 0.078). The area under the glucose curve was similar in both groups (*p* = 0.95), *i.e.*, the glycemic index of levan-enriched juice was lower than that of the regular one. 

**Table 3:** Results of mixed model for relative changes in gastrointestinal symptoms during 8 weeks of juice drinking.

Variable	Control group (*n* = 19)			Product group (*n* = 29)			Model effects (*p* values)		
	Baseline	Week 4	Week 8	Baseline	Week 4	Week 8	Treatment	Time	Treatment × time
Number of stools per week	9.37 ± 5.6	11.25 ± 6.15	9.82 ± 3.85	7.76 ± 3.23	9.09 ± 4.75	8.37 ± 3.82	0.14	0.02 *	0.79
Number of solid stools per week	4.84 ± 4.25	3.56 ± 4.86	3.51 ± 4.67	3.62 ± 2.69	3.17 ± 2.76	3.13 ± 2.69	0.41	0.11	0.84
Number of semi-solid stools per week	4.53 ± 6.04	6.96 ± 6.58	6.02 ± 5.29	3.9 ± 4.3	4.61 ± 5.09	4.98 ± 5.14	0.4	0.023 *	0.11
Number of fluid per week	0 ± 0	0.75 ± 1.29	0.39 ± 0.89	0.07 ± 0.26	1.33 ± 3.32	0.7 ± 1.63	0.27	0.01 *	0.85
Abdominal pain score	1.16 ± 2.69	1.32 ± 2.04	1.63 ± 3.23	0.9 ± 2.81	2.06 ± 3.14	1.41 ± 3	0.76	0.49	0.42
Bloating score	0.95 ± 3.29	2.75 ± 3.54	3.11 ± 5.75	2.9 ± 4.71	4.03 ± 5.64	2.78 ± 5.28	0.37	0.05 *	0.39
Vomiting score	0 ± 0	0.05 ± 0.23	0 ± 0	0 ± 0	0.03 ± 0.19	0 ± 0	0.99	0.18	0.89
Heartburn score	0.11 ± 0.46	0.68 ± 1.84	0.65 ± 1.77	0.45 ± 2.06	1.53 ± 3.53	0.86 ± 2.34	0.43	0.16	0.61
Gasses score	0.53 ± 0.51	0.28 ± 0.36	0.42 ± 0.43	0.38 ± 0.49	0.23 ± 0.36	0.36 ± 0.41	0.37	0.01 *	0.93
Epigastric pain score	0.89 ± 2.33	1 ± 2	1.23 ± 3.12	0.28 ± 1.16	0.44 ± 1	0.79 ± 0	0.09	0.98	0.68

* Significant difference between baseline and weeks 4–8.

**Table 4:** Results of mixed model for relative changes in BMI, blood pressure and blood tests during 8 weeks of juice drinking.

Variable	Control group (*n* = 19)			Product group (*n* = 29)			Model effects (*p* values)		
	Baseline	4 weeks	8 weeks	Baseline	4 weeks	8 weeks	Treatment	Time	Treatment × time
BMI	23.8	24.8	24.9	24.3	24.5	24.7	0.71	0.16	0.79
Systolic pressure	119.5	112.4	113.5	116.8	114.6	111.8	0.90	0.04 *	0.27
Diastolic pressure	74.8	69.8	70.3	74.4	71.8	72.7	0.34	0.03*	0.67
Hemoglobin (g/dL)	13.8	13.7	13.7	13.9	13.7	14.1	0.63	0.72	0.66
MCV (fL)	88.3	88.8	89.5	86.9	87.2	87.4	0.14	0.20	0.74
Glucose (mg/dL)		80.6	79.5	79.9	81.4	80.4	0.81	0.84	0.49
HBA1C (%)	5.4	5.3	5.3	5.4	5.3	5.6	0.61	0.78	0.64
BUN (mg/dL)	14.7	13.9	13.7	14.5	13.1	13.7	0.64	0.11	0.75
Creatinine (mg/dL)	1.0	0.9	1.0	1.0	1.6	0.9	0.84	0.77	0.003**
GOT (u/L)	21.4	23.2	21.6	20.8	20.6	23.0	0.73	0.66	0.12
GPT (u/L)	21.2	23.0	23.0	19.8	19.9	24.1	0.68	0.36	0.36
GGT (u/L)	17.9	18.1	17.9	17.4	15.5	16.9	049	0.60	0.33
Alk Phos (u/L)	66.2	66.9	68.5	62.8	62.8	65.8	0.23	0.11	0.64
LDL (mg/dL)	102.1	96.7	97.9	104.8	100.9	100.9	0.47	0.31	0.97
HDL (mg/dL)	55.3	57.7	58.3	59.6	58.9	61.1	0.39	0.50	0.39
Cholesterol (mg/dL)	174.1	171.8	174.7	182.4	179.6	181.4	0.25	0.60	0.95
Triglycerides (mg/dL)	83.2	87.3	91.4	87.2	93.8	97.5	0.62	0.49	0.94
Total bilirubin (mg/dL)	0.7	0.7	0.7	0.7	0.6	0.7	0.95	0.72	0.048 **
Transferrin (mg/dL)	224.4	234.0	283.9	224.8	232.9	233.0	0.80	0.30	0.93
Ferritin (ng/mL)	67.9	62.5	57.4	73.0	88.5	94.1	0.27	0.77	0.27
Sodium (mmol/L)	139.9	138.9	140.7	140.7	140.2	141.0	0.10	0.009 *	0.38
Potassium (mmol/L)	4.2	4.2	4.2	4.2	4.2	4.4	0.49	0.33	0.22
Chloride (mmol/L)	105.8	105.1	104.7	104.8	104.8	104.6	0.30	0.31	0.50
Phosphor (mg/dL)	3.5	3.3	3.5	3.6	3.5	3.4	0.82	0.19	0.22
Calcium (mg/dL)	9.1	8.9	9.2	9.2	9.0	9.2	0.45	0.0005 *	0.91
Magnesium (mg/dL)	2.2	2.2	2.2	2.2	2.2	2.1	0.88	0.21	0.49
Zinc (mcg/dL)	78.8	79.1	75.5	80.9	81.9	83.3	0.19	0.93	0.51
Albumin (g/L)	43.5	43.4	43.8	44.2	42.9	43.5	0.92	0.16	0.18
Iron (mcg/dL)	90.3	91.0	89.1	99.5	76.3	90.7	0.97	0.12	0.15
TSH (mu/L)	1.7	1.9	1.8	2.0	1.9	2.0	0.42	0.98	0.51

* Significant difference between baseline and weeks 4–8; ** Significant difference in interaction.

**Table 5:** Glycemic index of levan-enriched and regular juice.

Volunteer	Area under glucose curve		Area under insulin curve			
	Regular juice	Juice with levan	Regular juice		Juice with levan	
1	171	148.25	30.25	37.25		
2	164.75	169.25	62	44		
3	192.25	208.75	75.5	64.75		
4	181.5	173	48.25	29		
5	194	202.25	47.25	31.75		
*p* value	0.956		0.078			

## 4. Discussion

The results of the double-blind controlled study on healthy volunteers of 8 weeks administration of natural orange juice fermented with levansucrase (reduced sugar percentage and increased fiber content) *versus* regular natural orange juice showed no significant impact of levan on weight, gastrointestinal symptoms and metabolic profile. 

Levan-containing orange juice also caused no adverse events. Both regular and sucrose-reduced levan-enriched orange juices, however, decreased systolic and diastolic pressures, increased sodium levels (within normal range), increased stool number, and bloating scores, and decreased gas scores. This indicates that these effects were induced by the orange juice itself and not by the reduction of sugar or the increase in fiber content. The participants in the study group followed an 8-week diet that was almost double the fiber content than that of the controls (22.45 g *versus* 13 g) and than their pre-study diet (22.45 g *versus* 11.2 g). This change in fiber content had no effect on either the metabolic profile or gastrointestinal parameters.

Many studies that emerged during the past decades described a benefit of dietary fiber intake, such as a decreased risk of colorectal cancer, [10] lowering of cholesterol and triglycerides levels [11], and relief of irritable bowel syndrome symptoms [12]. Fibers which can be fermented by intestinal flora (prebiotics) are of special interest. Inulin, which, like levan, is a member of the fructans family, has a well-known prebiotic effect [2,5,13]. It has a proven hypo-triglyceridemic effect [13,14,15], an ability to enhance mineral absorption [3], and even a possible effect of reducing the risk of colon cancer [10]. Unlike inulin, levan has not been investigated in depth. Some in vitro studies showed that all beta-fructans (including levan) are fermented by intestinal bacteria and can be classified as pro-biotics [5], but levan has not been classified as a prebiotic fiber since in vivo studies on rats [7] did not support the proposition that it can be fermented by strains of bifidobacteria. We did not study the prebiotic characteristics of levan in colonic and stool cultures, but we did expect some impact of levan on gastrointestinal symptoms, which was not shown by our results. One possible explanation is that the usual Israeli diet has a relatively high fiber content as we saw in the pre-study diets of both groups. The findings of the current study showed a significant decrease in the blood pressure (within normal range) as result of orange juice consumption in normotensive subjects. Although it is a common belief that orange juice is able to lower blood pressure in hypertensive patients, we believe that this is the first controlled study to look into this subject. Based on the findings of our study, further research into the impact of orange juice on hypertension is warranted. 

A very interesting question is the possible effect of sugar-induced juice on glucose control. Our study showed that despite a lower glycemic index in the pilot group, no influence of the juice on glucose level and HBA1C was demonstrated in either group. However, both groups included only healthy volunteers. Therefore the possible effect of sugar-induced juice on glucose control should be studies on diabetic patients or patients with border-line hyperglycemia. 

The results of Yamamoto *et al.*’s study using a rat model suggested a hypocholesterolemic effect of levan [7]. The study of Kang *et al.* showed some positive effect of levan on weight, body fat, cholesterol profile and triglyceride level in Korean women [9]. Our current study showed no similar effect of levan-supplemented or plain orange juice on the levels of cholesterol or triglycerides among healthy subjects with normal cholesterol and triglycerides levels. Further studies on the possible effects of orange juice alone and orange juice enriched with levan on hypertensive and hyperlipidemic patients might yield interesting findings. Also interesting is the possible effect of fiber on mineral absorption, as suggested by past animal studies [4]. We found effects of levan on blood levels of any mineral (calcium, magnesium, iron, *etc.*). Finally, the sodium blood levels increased significantly in both our study and control groups (within normal limits). Since there were no differences between the groups, the effect must have been due to the orange juice itself. 

## 5. Conclusion

In summary, we found that daily consumption of a fiber-enriched, sugar-reduced orange juice for 8 weeks caused no significant impact on metabolic parameters and gastrointestinal symptoms in healthy participants compared to regular orange juice. Further studies on the effect of levan on obese, hypertensive and hyperlipidemic patients and on the possible effect of orange juice itself are warranted. 
